# Oscillatory Threshold Logic

**DOI:** 10.1371/journal.pone.0048498

**Published:** 2012-11-16

**Authors:** Jon Borresen, Stephen Lynch

**Affiliations:** School of Computing, Mathematics and Digital Technology, Manchester Metropolitan University, Manchester, United Kingdom; National Microelectronics Center, Spain

## Abstract

In the 1940s, the first generation of modern computers used vacuum tube oscillators as their principle components, however, with the development of the transistor, such oscillator based computers quickly became obsolete. As the demand for faster and lower power computers continues, transistors are themselves approaching their theoretical limit and emerging technologies must eventually supersede them. With the development of optical oscillators and Josephson junction technology, we are again presented with the possibility of using oscillators as the basic components of computers, and it is possible that the next generation of computers will be composed almost entirely of oscillatory devices. Here, we demonstrate how coupled threshold oscillators may be used to perform binary logic in a manner entirely consistent with modern computer architectures. We describe a variety of computational circuitry and demonstrate working oscillator models of both computation and memory.

## Introduction

In 2005, the US National Security Agency [1] concluded that transistors were rapidly approaching the limits of functionality and that new technologies needed to be developed in order to overcome this.

Recent advances in novel computation suggest a variety of new computing technologies which may be applicable and have far reaching applications. Of particular note are: quantum computing [2]–[6], all-optical computing [7]–[11], spin computing [12]–[15], chaos computing [16]–[18], and DNA computing [19]–[23]. Each of these suggests a technology which is in many respects fundamentally different from current computing systems and in order for their implementation to be successful, new programming structures and techniques would be required, in addition to any development of reliable working prototypes.

One technology which is considerably more advanced from an implementation perspective is superconductive computing [24], [25], based on Rapid Single Flux Quantum (RSFQ) technology [26]–[30] which effectively uses Josephson Junctions (JJs) to replace transistors as the fundamental active element in any circuit. This technology is more mature than the others mentioned above and has already produced practical digital and mixed-signal circuits with world record breaking processing speeds at exceptionally low power [31]–[36]. The RSFQ-based digital receivers were demonstrated in the field, converting high-frequency wide-band analog communications signals to digital domain taking advantage of extreme sampling speeds of RSFQ circuits [37]–[41]. The JJ RSFQ circuits are fabricated using a relatively simple thin-film process developed in several places [42]–[46]. Recently, a new energy-efficient generation of RSFQ circuits has been introduced [47] complemented with energy-efficient cryogenic memory [48] leading to the implementation of energy-efficient computing.

Neurons can also be simulated using simple JJ circuits [49]–[53]. These circuits could then be connected to form logic gates similar to those appearing in this paper. The NSA report [1] also concluded that the most likely successor to transistor technology would be JJ circuitry. The authors are currently investigating implementation of binary logic using neuronal JJ circuitry and the results will be published at a later date.

One further technology which is somewhat less advanced from an implementation perspective is neuronal computing (or wetware), which uses artificially grown neurons as the processing units. Again such technology would require a considerable shift in how computers were designed and programmed and is unlikely to prove to be a successor to Complementary Metal-Oxide-Semiconductors (CMOS) at any time in the foreseeable future. What neuronal computing does provide is a deeper understanding of brain functionality and possible associated medical benefits that would therefore follow. Recently, it has been reported that scientists were able to grow brain nerve cells affected by Parkinson's disease using human skin cell samples [54]. These neurons could be connected to form logic circuits similar to those reported in this paper. Degradation of logical functionality could then be used as an assay to determine the effect of drugs or physical damage on neuronal circuitry.

Zanin et al. [55], show that computation can emerge from collective dynamics of an ensemble of networking neurons. Synchronization and desynchronization of neurons using a dynamic weighted network is used to codify binary information. Additionally, neural encoding using conjugate symmetries [56] and the concept of ‘winnerless competition’ in coupled oscillator networks [57] have also been demonstrated as viable methods of oscillatory computation.

Threshold logic [58], [59] and null convention logic [60] have also been considered, particularly with respect to neural network computing. Beiu et al. [61] provide a review of commercial VLSI hardware implementation of threshold logic up to 2003. With the emergence of nanotechnologies; resonant tunneling, single electron and memristor implementation are also looking promising, especially with respect to energy delay efficiency and reconfigurable circuits [62]–[67]. Additionally, Modified Variable Threshold Logic (MVTL) using JJs has demonstrated high speed, low power processing [68].

In this paper, we demonstrate a novel computational concept using both inhibitory and excitatory connections between threshold oscillators. The use of inhibitory connections in this way draws on ideas from biological neural encoding and has not previously considered as a tool in implementing binary computation. The use of threshold logic in oscillator circuits presents numerous opportunities for novel circuit design. The inherent richness of oscillator dynamics allows for excitatory and inhibitory connections which may be in many respects different from standard threshold weights used in traditional threshold logic: Neuronal circuits are known to display phase and anti-phase synchronization [69] which may have applications in clocking and error correction. Bursting type behavior [70] could be utilized to prevent signal degradation and improve noise resilience. Connections may induce a variety of responses [71] - all or nothing, additive, amplitude or frequency, which may have specific uses in circuit design.

The method by which inhibition may be caused to occur would be specific to the type of oscillator or oscillatory circuit in question. Neural inhibition has been widely studied [72]. In biological neural circuits, excitation and inhibition occur via diffusion of neurotransmitters across a synaptic gap. For inhibition, this has a temporal effect which permits suppression of post synaptic neural activity for specific time intervals (known as the *refractory period*). In this paper, we use a method of inhibition using negative connection weights similar to that employed in standard threshold logic design, however, this is to demonstrate proof of principle and is not the only possible method by which logic gate connections could be made. Electrical circuit oscillators can likewise demonstrate inhibition [73]. Optical oscillator circuits offer the possibility of interference based inhibition [74] and perhaps most importantly, given recent advances in JJ technology, JJ oscillator circuits have also been designed with inhibitory characteristics very similar to those in neural circuitry [52].

The underlying concept is not technology specific and, given recent advances in JJ circuitry and neuronal computing, could be readily implemented [75], [76]. It is also considerably less disruptive in that the underlying binary logic is identical to that employed in CMOS and implementation in the JJ form would be compatible with traditional computing architectures.

We present schematics for simple arithmetic and memory operations and describe these operations as the solution set of a system of linear inequalities. Simulations using a neural oscillator model demonstrate how such a model may be implemented.

## Methods

Computing using oscillators is not a new concept, indeed the first modern computers were made using vacuum tube oscillators, and oscillators in a variety of forms are integral components in many devices. The use of neural oscillators has also been widely studied, however, in all cases the method of computation is derived from concepts of biological neural encoding. Current research into encoding using neural oscillators is therefore spatio-temporal, rate, or more usually synchronization based [77]–[79].

What has not previously been considered is using oscillators as the fundamental components of computing devices (with all the inherent dynamical richness that this provides) and designing them in such a way as to perform binary logic in an equivalent manner to standard transistor logic - that is the oscillator will provide a binary output (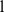 equivalent to an oscillator firing or 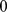 where the oscillator does not fire) and the output from a single oscillator can be interpreted in exactly the same way as that of a transistor.

### Threshold logic

Threshold logic has been studied as an alternative to Boolean logic for some time. For many implementations this is advantageous, allowing for reduced component counts and/or number of logic levels, as the implementation of complex logical operations may be achieved using a single gate [80].

Threshold logic gates [81] have a set of inputs 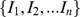, weights 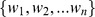 and a binary output 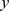. The output 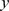 is typically described by:
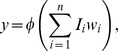
where the function 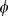 is an activation function (eg Heaviside, tanh, sigmoid, piecewise linear, low gain saturation [82]) and the binary output 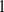 is defined at some threshold 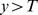, say.

Threshold logic implementation has not supplanted standard logic implementation in CMOS due to sensitivity to parameter changes and variable connection weights requiring very low tolerance engineering. Recent advances in nanotechnology, in particular, Resonant Tunneling Devices (RTD) [83] and memristor devices [84] have the potential to overcome such concerns.

### Generic threshold oscillator model

A threshold oscillator is an oscillatory device that will begin oscillating when the input to the device is above a certain threshold. Below this level the oscillator remains in a resting state and gives no output. It is possible to use the output of one threshold oscillator as the input of another oscillator to cause the second oscillator to operate (excitation) and under certain circumstances, it is also possible to cause the input of one oscillator to suppress the output of another oscillator (inhibition).

There are numerous viable methods for implementing binary computation using threshold oscillators. In order to perform the logical operations it is necessary that either oscillators with differing thresholds be used or the connections to the oscillators be of differing weights. In our modeling we shall use the latter method as this mimics more closely biological neural systems, from where the idea originated.

### Logical operations

Logical operations can be performed in a similar manner to standard logic circuits, however, due to the threshold nature it is possible to formulate logical operations as solutions of sets of linear inequalities. For instance, the AND function can be replicated by a threshold oscillator with two inputs, where the input strengths are scaled such that the total input is only above threshold if both the inputs are on. For a single input or for no input the total input would be below threshold. Defining the inputs to the logical circuits in vector form and scaling the input strength to a binary 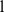 or 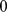, we write 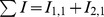 as the total input to the circuit. The threshold equations may be thus written as:
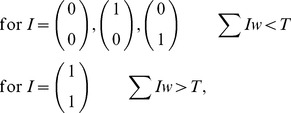
(1)where 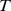 is the oscillator threshold and 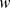 the coupling weight between the inputs and the oscillator performing the AND operation. Clearly the solution to the above system Eq. (1) is 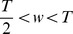. For the logical OR operation, the solution 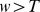 would suffice.

Using threshold oscillators in this manner it is straightforward to implement the logical NOT operation using a negative coupling strength, however, as the logical NOT is effectively redundant in more complex logically complete circuit design where NAND and XOR operations are used, we will present all models using the latter formulations.

### Binary half adder

**Figure 1:**
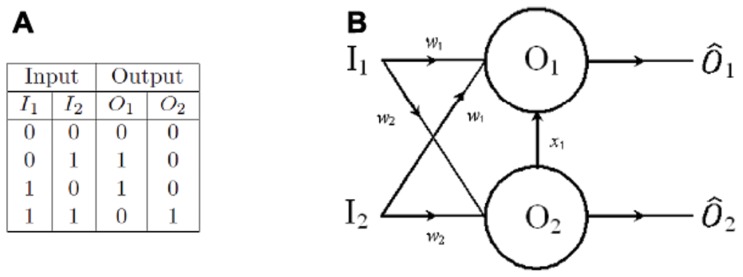
Binary half adder. (A) Truth table for a binary half adder. (B) Oscillator circuit diagram for a binary half adder comprising two inputs 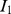 and 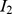 and two oscillators 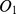 and 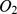. The sum oscillator 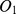 will oscillate if either 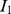 or 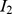 are active. The carry oscillator 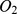 will oscillate if both 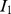 and 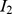 are active. An inhibitory connection from 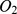 to 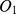 suppresses oscillator 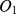 if 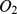 is active.

One of the simplest computing circuits is the binary half adder. The binary half adder gives the sum of two binary inputs as a two bit binary output. The truth table for the binary half adder is given in Fig. 1A.

Standard transistor implementation of a binary half adder uses one XOR gate (to give the sum) and one AND gate (to give the carry). Implementation of this circuit using threshold oscillators can be achieved via a similar design, with two oscillators replicating the logical functions. The AND operation is implemented as described above and the XOR operation can be achieved using an OR operation (as above) with an additional connection from the AND oscillator, which in some way inhibits the operation of the OR oscillator if the AND oscillator is active. The method by which inhibition occurs would be dependent upon the oscillators being used to form the circuitry.

Fig. 1B demonstrates a viable circuit schematic for half adder implementation using two oscillators 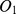 and 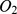 and two inputs 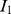 and 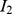, which may themselves be the output from other oscillators in a more complex circuit. Schematically, the circuit design is not dissimilar to standard threshold logic half adders [58], however, due to the nature of the connections between oscillators, implementation may be markedly different. If we consider oscillators with identical thresholds we will require that the coupling strength, 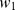, say, from 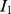 and 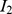 to 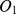 be sufficient to cause 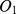 to oscillate for only one input and for the coupling strength, 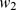, say, from 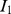 and 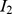 to 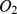 to be sufficient for it to oscillate for two inputs. The additional connection 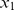, say, from 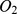 to 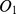 is inhibitory such that if 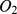 is oscillating it suppresses 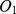. Denoting the output from 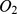 as 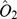, the total input to 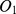 and 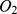 are thus given by:
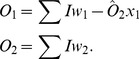
(2)


We can consider such a system as a set of linear inequalities with normalized input vectors 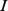 and threshold 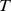 requiring solutions of the form:
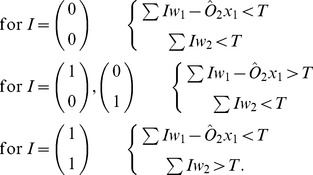
(3)


Thus, for instance, for a total input of 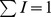, only 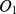 will be above threshold causing oscillation giving a binary equivalent output of 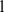. If both 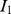 and 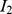 are active, 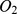 will oscillate but 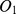 is suppressed if 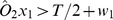, giving a binary output 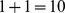, as required.

### Two-oscillator full adder

**Figure 2:**
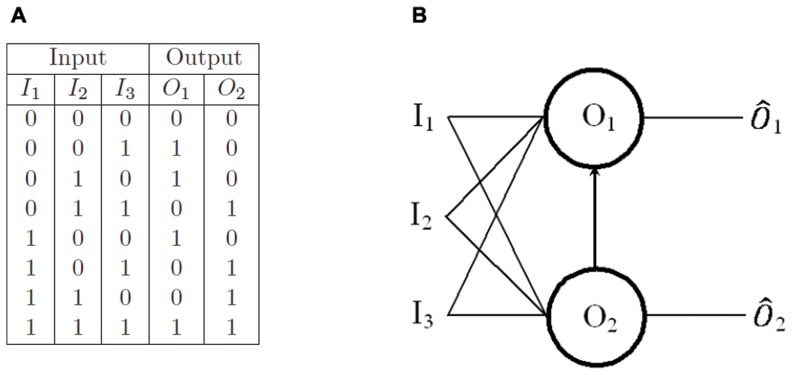
Binary full adder. (A) Truth table for a binary full adder. (B) Oscillator circuit diagram for a binary full adder comprising three inputs 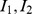 and 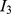 and two oscillators 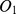 and 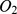. Oscillator 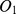 will oscillate if either 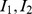 or 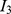 are active. Oscillator 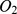 will oscillate if any two of 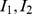 and 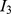 are active. An inhibitory connection from 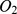 to 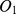 suppresses oscillator 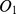 if 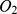 is active, however, the inhibition is only sufficient to suppress 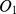 for 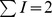. For inputs of 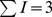 the total input to 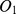 is still sufficient to induce oscillation.

In order to demonstrate how oscillatory threshold logic scales for operations on larger numbers of bits we will consider the next simplest arithmetic circuit - the binary adder (or full adder). The binary adder has a truth table as in Fig. 2A.

Using traditional circuitry the binary adder requires five logic gates to perform such an operation. It is possible to formulate the adder circuit using oscillators as a solution set of two linear inequalities and as such only two oscillators are required to perform the operation. The oscillator equations are the same as Eq. (??) and the threshold inequalities are given by:
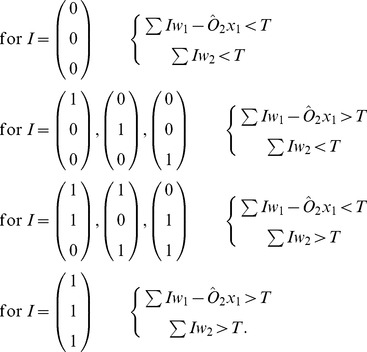
(4)


In keeping with other full adder threshold logic designs [58], [85], the full adder schematic (Fig. 2B) is essentially identical to those of the half adder except for the additional input 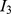.

It is possible to use additional oscillators to give the the binary sum for 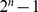 inputs using only 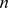 oscillators. Each additional oscillator acts as the next binary digit of the required output and the respective input weights are adjusted thusly. Inhibitory connections from the additional oscillator to all other oscillators are required such that the inhibition strength from each new oscillator is scaled accordingly. Thus an exponential increase in computational power could (theoretically) be provided by a linear increase in the number of fundamental components. For larger circuits, the number of interconnections within the circuit would increase considerably, as the computation is effectively encoded in the connections rather than the switches themselves. Given sufficient engineering capability to provide the necessary connectivity, the number of components in any circuit and the time required to perform calculations - as a function of the required switching time of the oscillators - could be considerably reduced in this way.

## Results

### The Fitzhugh-Nagumo model

The Fitzhugh-Nagumo system [86], [87] is one of the more well known oscillator models. It is essentially a reduction of the Hodgkin-Huxley equations [88] which describe the action potential of a spiking neuron. The model is fairly straightforward to implement as an electrical circuit, as demonstrated by Binczak et al. [73].

The describing equations are:
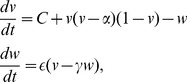
(5)where 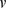 is a fast variable (in biological terms - the action potential) and 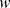 represents a slow variable (biologically - the sodium gating variable). The parameters 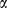, 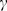 and 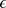 dictate the threshold, oscillatory frequency and the location of the fixed points for 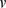 and 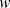. The model will begin to oscillate when the input current 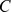 is above a critical threshold 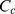. For all the following simulations, the threshold 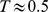.

It is possible to couple the oscillators together via various methods. For biological neural systems, where there is synaptic coupling between neurons the coupling function is complex, relying on diffusion of neurotransmitters across a synaptic gap. The connections between neurons may either depolarize (excite) or hyperpolarize (inhibit) the post synaptic neuron.

Crucially, the hyperpolarizing inhibitory effect has a temporal component such that if inhibition occurs, the post synaptic neuron remains inhibited for some period of time after the pre-synaptic neuron fires. It is not straightforward to simulate such a system using the Fitzhugh-Nagumo model without either integration of the signal pulse or introducing arbitrary conditions on oscillators receiving an inhibitory pulse - which would not be viable from an implementation perspective. As such we will employ a method which is phenomenologically similar to neural hyperpolarization but is not necessarily consistent with any biological process.

**Figure 3:**
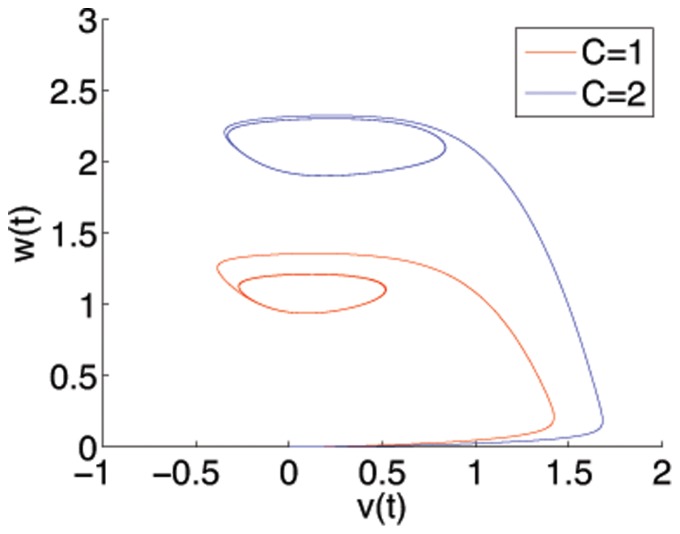
Phase portrait for Fitzhugh-Nagumo model with 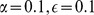 and 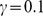 shown for two input strengths 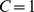 and 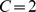. From an initial resting state at 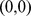, the transient dynamics is of a wide trajectory before settling onto a limit cycle.

**Figure 4:**
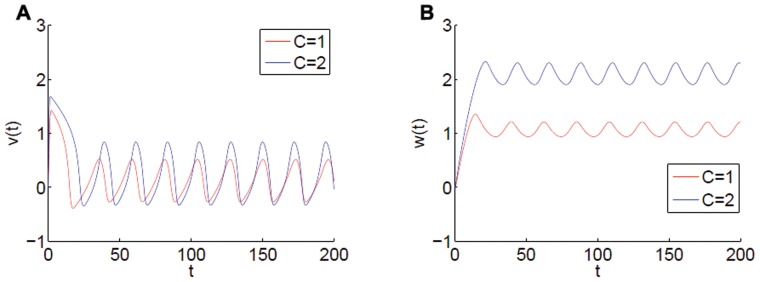
Associated time series of 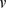 and 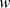 for corresponding Fig. 3. Note that after transients, (A) 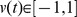 for 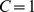 and 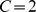, (B) whereas 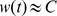.

Implementation by coupling through either the fast 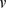 variable or the slow 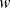 variable are equally viable. As can be seen from Fig. 3 and Fig. 4 for varying inputs 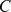, the fast voltage oscillates with a fairly constant maximum and minimum whilst the slow variable 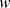 oscillates around a fixed point at approximately 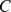. Any coupling function to be used must take into account the specific dynamics of whichever variable is used.

As is common in such biologically inspired models we will use a sigmoidal transfer function between oscillators of the form:
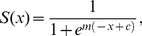
(6)where 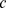 is the threshold at which the output begins to rise and 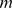 denotes the steepness of the curve of the function 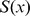. In biological systems, neural connections can exhibit plastic responses and become ‘tuned’ (via some Hebbian learning rule [89]) allowing for more reliable excitation and inhibition. Choosing suitable values of 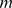 and 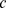 would in many respects replicate such a process.

### Fitzhugh Nagumo half adder and full adders

Numerical simulations for systems of Fitzhugh Nagumo oscillators coupled as in Figs. 1B and 2B will now be discussed. It is also shown that by adding one more oscillator to the two-oscillator full adder it is possible to construct a three-oscillator seven-input full adder.

The inputs to the logical circuits are oscillatory, being provided by Fitzhugh-Nagumo oscillators with similar coupling and parameter values to the computational oscillators. Oscillatory inputs of this form have been chosen over continuous inputs, as this demonstrates the necessary robustness of signal integrity which would be required for larger computational circuits. Continuous inputs to the computational oscillators would be equally viable and present no difficulties in implementation. As such the matrix form for the input weights for each oscillator is 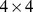 rather than 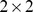 as two additional oscillators are used as inputs.

**Figure 5:**
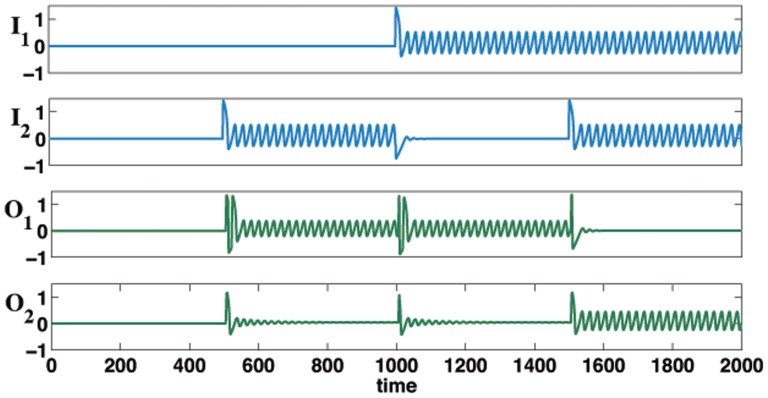
Time series for Fitzhugh-Nagumo binary half adder. The parameter values taken for the transfer function 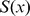, see Eq. (5), are 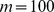 and 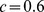. All binary combinations of oscillatory inputs 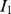 and 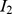 give the required binary outputs 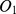 and 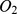 (see Fig. 1A). Here 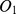 represents the sum and 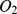 the carry in terms of standard logical circuitry. The observed pulse like behavior after switching is due to the wide trajectories taken by the input oscillators after being perturbed from the resting state.

One solution, in matrix form, to the inequalities Eq. (2) and Eq. (3) for the binary half adder, would be:
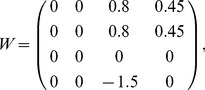
(7)where 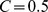 in Eq. (5) for the inputs 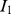 and 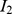. This would give the parameter values in Fig. 1B as 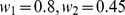 and 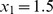. The time series for such is shown in Fig. 5.

**Figure 6:**
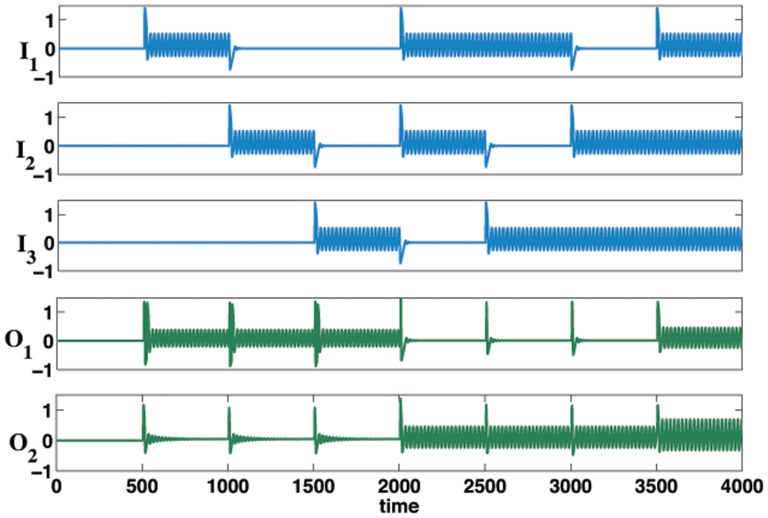
Time series for a Fitzhugh-Nagumo two oscillator full adder. All binary combinations of oscillatory inputs 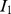, 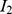 and 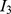 give the required binary outputs for 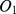 and 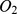 (see Fig. 2A). The weights used in this example were again 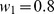, 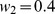 and 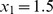 (see Eq. (4)).

A two-oscillator binary full adder can be constructed by simply introducing another input, 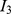, say, as in Fig. 2B. Fig. 6 shows the time series for the Fitzhugh-Nagumo two-oscillator full adder.

**Figure 7:**
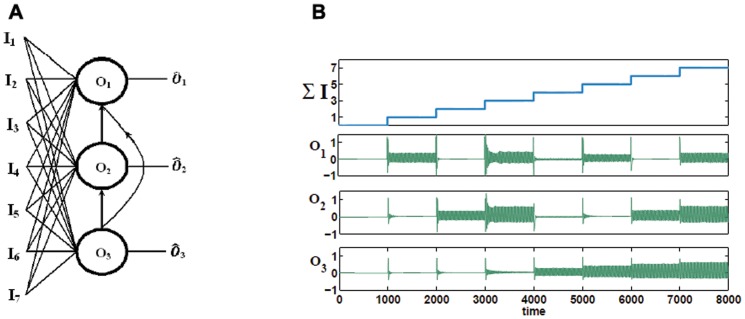
Seven input, three oscillator full adder. (A) Block diagram for seven input, three oscillator full adder. (B) Time series for a Fitzhugh-Nagumo three-oscillator full adder. The total input strength 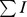 is shown in the first row. All binary combinations of oscillatory inputs 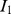 to 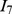 give the required binary outputs for 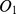, 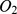 and 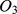. The weights used in this example were 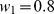, 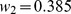, 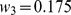, 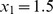, 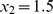 and 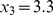, where 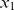 is the inhibitory connection from 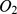 to 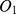, 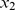 is from 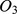 to 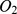 and 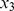 is from 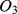 to 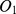. Note that the amplitude of oscillation increases when the sum of the input to any oscillator is greatly above threshold, however, the binary on/off response is as required.

To conclude this section, we consider a three-oscillator seven input full adder (see Fig. 7). Fig. 7A shows a schematic of the three-oscillator seven input adder comprising twenty one excitatory connectors and three inhibitory connectors. Fig. 7B shows the time series of the seven input three-oscillator full adder.

### Fitzhugh Nagumo 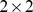 bit binary multiplier

In order to more fully demonstrate the applicability of such a model we have also simulated a more complex circuit, namely a 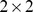 bit binary multiplier. Such a circuit outputs the binary multiple of two, 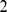 bit binary inputs. Although it is possible to perform such a calculation using only four oscillators we have used a standard circuit implementation of a binary multiplier to demonstrate how oscillators could be used to replace transistors as the fundamental units of computing devices without the need for architectural redesign.

**Figure 8:**
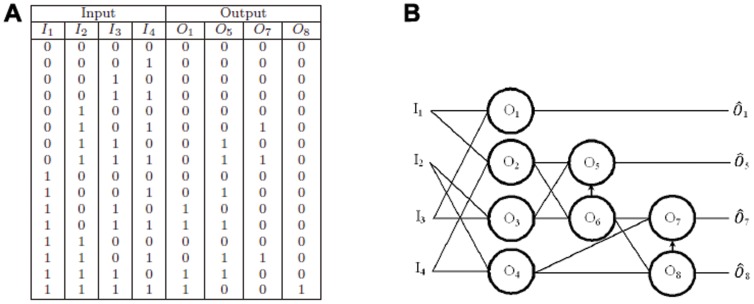
A 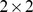 bit multiplier. (A) Truth table for 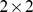 bit binary multiplier. (B) Schematic of the binary oscillator 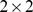 bit multiplier using oscillators based on standard circuitry.

**Figure 9:**
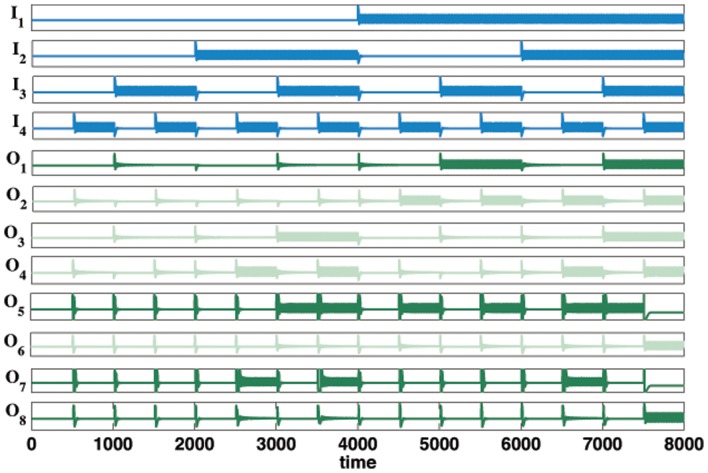
Time series of a 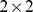 bit multiplier using oscillators based on standard circuitry. All possible binary combinations for inputs 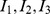 and 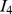 are shown. The oscillators 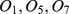 and 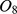 represent binary digit representation of the powers of 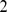. Oscillators 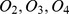 and 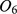 are used in the internal circuitry only and their time traces have been made fainter to emphasize the binary output oscillators. For example, at time 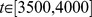 the inputs 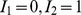 representing the decimal 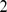 and 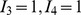, representing the decimal 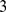, giving an output where 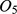 and 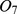 are oscillating, being the binary equivalent 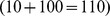 of the decimal 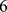. Note 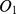 and 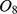 are not oscillating. This is equivalent to column eight above and row eight in the truth table Fig. 8A.

Fig. 8A shows the logic table for a 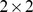 bit binary multiplier and Fig. 8B shows the corresponding schematic of the oscillator circuit. The time series of the 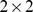 bit binary multiplier is displayed in Fig. 9.

### Fitzhugh Nagumo set reset flip-flop

**Figure 10:**
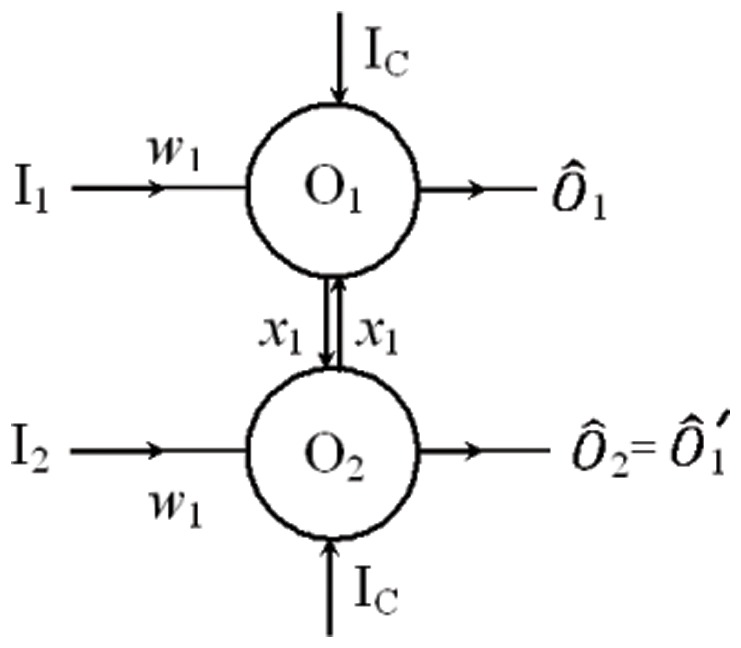
Schematic of an SR flip flop using oscillators. Both oscillators 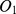 and 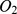 receive an external input current 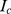 and there are inhibitory connections 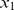 between the oscillators. From an initial state where one oscillator is active the other will remain suppressed. External inputs 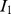 or 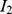 to the inactive oscillator will induce a switch. The oscillators are effectively performing NOR logical operations as in a conventional SR NOR latch.

**Figure 11:**
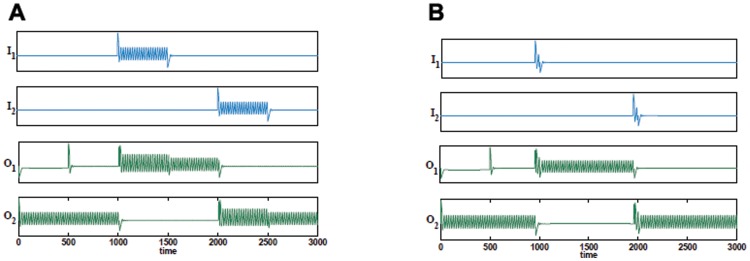
Time series of an SR flip-flop using oscillations to switch. (A) Simulation with 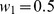 and 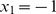. The simulation is initialized using a single external current to 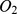 for 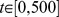. At 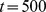, oscillator 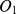 also receives an external input current, however, it is suppressed by the output from 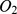. The initial state has 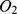 active and 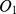 inactive. A continuous switching pulse is provided by 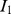 at 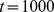. At 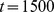, this switching pulse is turned off, but 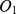 and 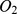 remain in the switched state (as required). A further switch is performed at 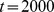 using a continuous pulse to 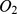. (B) Time series of an SR flip-flop using single input pulses to switch, with 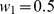 and 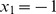. The switching is performed as for case A, however, only one pulse cycle (ballistic propagation) is used. Note the switching occurs as required and the system remains switched once the pulse has been received.

Fig. 10 shows a schematic of a Set-Reset (SR) flip-flop circuit, the input 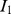 is commonly referred to as the Set and input 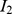 is referred to as the Reset. Output 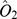 is the complement of output 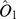. Note that both oscillators require a constant input 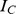, say, for the circuit to function properly. This circuit acts as a memory, storing a bit and presenting it on its output 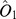, as can be seen in Fig. 11.

The SR flip-flop described here is an application of the ‘winnerless competition’ principle [57]. In the absence of coupling between the oscillators, both will remain active. However, a symmetric inhibitory coupling between them ensures that from an initial state, where only one oscillator is active, the other will remain suppressed in the absence of any external perturbation. When an input is given to the inactive oscillator this is switched on, simultaneously suppressing the previously active oscillator. When the external input is turned off, the system remains in the switched state. Note that for a switch to occur, an input pulse of only one period is required (see Fig. 11). Switching using a single pulse in this way can open an opportunity to use ballistic propagation of signals between gates and memory cells, which could significantly reduce the energy required to operate memory circuits, where currently power intensive line charging is required to initiate memory switches.

**Figure 12:**
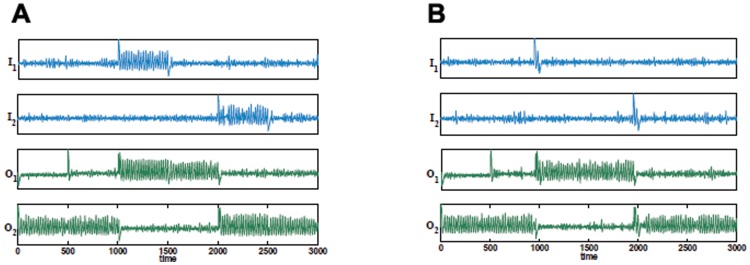
Controlled switching in the presence of noise. (A) and (B) Time series of SR flip-flop as in Fig. 11 with additional Gaussian noise, mean = 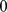, standard deviation = 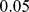, added to all oscillators. In both cases the switching occurs as required.

One important consideration, particularly with respect to flip-flop circuits is the ability to switch accurately in the presence of noise [90]. A detailed examination of error rates arising from such noise would be specific to the oscillator and the underlying circuit implementation. For the general case considered here (a Fitzhugh-Nagumo implementation) repeated simulations have demonstrated that the system is particularly resistant to such noise (see Fig. 12) as is often the case with coupled oscillator dynamics [91], [92]. As the oscillators in the flip-flop circuit will only ‘switch’ if provided with a pulse above threshold, for a noise induced switch to occur (an error) it would be required that the noise amplitude itself be above this threshold. Although such an error is conceivable, no such error has been observed for Gaussian white noise with a standard deviation below 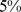 of the oscillator threshold. It should also be noted that in any JJ implementation of an oscillatory SR flip-flop, noise is effectively reduced to zero due to the required supercooling.

## Discussion

Here we have demonstrated how coupled threshold oscillators may be used as the principle components of the next generation of computers. Such implementation using binary logic is not disruptive, in that, from a programming and architectural perspective no significant changes are required.

There are clearly considerations concerning the accurate functioning of any such highly connected circuits, particularly with respect to multiple inputs to a single gate [93], [94], however, it should not be overly problematic to reduce the component numbers significantly given modern engineering capabilities.

The Fitzhugh-Nagumo simulations of the half adder, two-oscillator full adder, three-oscillator full adder, and 2×2 bit multiplier demonstrate threshold oscillators performing all the necessary components of arithmetic logic, while the SR flip-flop demonstrates the potential for very low power memory, particularly when ballistic propagation is considered.

Although the Fitzhugh-Nagumo models demonstrated here are in many ways phenomenologically similar to neural dynamics, we are not attempting to make any inference as to neural dynamics themselves. Moreover, we use the Fitzhugh-Nagumo system as an exemplification of the idea. In practice, implementation via Fitzhugh-Nagumo circuitry would be unlikely as this would offer very little in terms of speed or power consumption, however, any implementation via JJs or optical oscillators could be achieved in a very similar manner to that described for the Fitzhugh-Nagumo model whilst providing exceptional processing speed for minimal power usage.

There is currently a drive to low power exascale supercomputing (a computer which performs more than 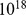 floating point operations per second (FLOPS)). The previous world's fastest supercomputer, the K computer, operates at a maximum performance of 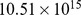 FLOPS and requires approximately 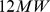 of power (excluding the power for the cooling system which is typically in excess of 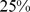 of power). This has recently been overtaken by the Sequoia IBM BlueGene/Q operating at 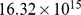 FLOPS and using 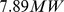 [95]. Even with such continued improvements in power consumption it is clear that without a significant technological breakthrough beyond that offered using standard CMOS transistor technology the prospect of an exascale computer is currently unviable. The use of oscillatory threshold logic presents a plausible avenue for implementation for which the engineering capability currently exists and which could be readily implemented.
